# Seroprevalence of Human Herpesvirus Infections in Newly Diagnosed HIV-Infected Key Populations in Dar es Salaam, Tanzania

**DOI:** 10.1155/2021/4608549

**Published:** 2021-08-25

**Authors:** Doreen Kamori, Agricola Joachim, Mucho Mizinduko, Godfrey Barabona, Macdonald Mahiti, Upendo Kibwana, Mtebe Majigo, Salim Masoud, Ambele M. Mwandigha, Takamasa Ueno, Elia Mmbaga, Eligius Lyamuya

**Affiliations:** ^1^Department of Microbiology and Immunology, Muhimbili University of Health and Allied Sciences, Dar es Salaam, Tanzania; ^2^Department of Epidemiology and Biostatistics, Muhimbili University of Health and Allied Sciences, Dar es Salaam, Tanzania; ^3^Joint Research Center for Human Retrovirus Infection, Kumamoto University, Japan; ^4^Department of Community Medicine and Global Health, University of Oslo, Norway

## Abstract

**Background:**

Human herpesvirus (HHV) infections can significantly increase the risk of human immunodeficiency virus (HIV) transmission and accelerate disease progression. In the population at high risk of HIV infection, also termed as key populations (female sex workers (FSW), men who have sex with men (MSM), and people who inject drugs (PWID)), and their sexual partners, HHV infections can potentially compromise the efforts to prevent and control HIV infection. Here, we investigated the seroprevalence of HHV infections among HIV-infected key populations in Dar es Salaam, Tanzania. *Methodology*. We analyzed 262 archived serum samples of HIV-infected key populations from the integrated biobehavioral surveillance (IBBS) study conducted in Dar es Salaam, Tanzania. The enzyme-linked immunosorbent assay was used to determine IgG and IgM titers for cytomegalovirus (CMV) and herpes simplex virus (HSV) types 1 and 2.

**Results:**

The overall seropositivity of HHV IgG was 92% (95% CI: 87.7–95.3%). HHV IgM was not detected in any of the samples. The most seroprevalent coinfection was CMV at 69.1% (181/262), followed by HSV-2 33.2% (87/262) and HSV-1 32.1% (84/262). HSV-2 infection differed by key population groups; it accounted for FSW (46.3%) (*p*=0.0001) compared to PWID (21.6%) and MSM (22.7%). In contrast, seroprevalence for CMV and HSV-1 was comparable across the key population groups; whereby, CMV was 62%, 75.3%, and 75% and HSV-1 was 26.4%, 39.2%, and 31.8% for FSW, MSM, and PWID, respectively. We also observed that multiple coinfections with CMV-HSV-2 (*p*=0.042) and CMV-HSV-1-HSV-2 (*p*=0.006) were significantly associated with key population aged above 40 years.

**Conclusion:**

The IgG seroprevalence of CMV, HSV-1, and HSV-2 was high among HIV-positive key populations. These findings indicate that these individuals are prone to recurrence of HHV infections and may harbor replicating viruses that subsequently may affect HIV disease progression. Therefore, this warrants concerted efforts for integrated HIV and sexually transmitted infection prevention programs targeting key populations.

## 1. Introduction

HIV key population groups (female sex workers (FSW), men who have sex with men (MSM), and people who inject drugs (PWID)) form a small percentage of people in sub-Saharan Africa (SSA) who are at high risk of HIV infection. The key populations FSW account to 4%, PWID 2%, and MSM 6% of the new infections in SSA [1]. In 2017, this group and their sexual partners contributed 17–40% of new HIV infections in the SSA region [1], highlighting the epidemiological importance of addressing key populations in the strategy to end HIV in the region by 2030 [2].

Human herpesvirus (HHV) and HIV infection have multiple bidirectional interactions [3]. HHV infections can significantly increase the risk of HIV transmission and accelerate disease progression [4, 5]. For example, HIV and HSV-2 coinfections associate with up to 0.5 log copies/ml increase in HIV plasma viral load [5], which increase transmissibility of HIV-1 infection and progression to acquired immunodeficiency syndrome (AIDS). In addition, HSV-2 genital ulcers tend to be extensive and persistent in HIV-infected individuals, increasing the transmissibility of HIV [6–8]. On the other hand, chronic infection of HHV-5, cytomegalovirus (CMV) has been associated with immunosenescence and immune activation in the general population [9], leading to immune exhaustion. CMV infection is more pronounced in HIV-infected patients and is considered a critical factor in sustaining immune activation. The end outcome links to non-AIDS complications among subjects on antiretroviral therapy (ART) [10]. The practice of HIV-risk behaviors among key populations also exposes them to an increased risk of HHV infections due to shared routes of transmission [11]. Therefore, HIV and HHV coinfection in key populations worsens HIV disease progression and can further fuel HIV transmission to sexual partners, PWID partners, and the general population.

In Tanzania, the prevalence of HIV among key populations is 3–6 times higher than that of the national average [1, 12–14], making this group the most affected and important reservoir for HIV transmission to the general population. However, data on the seroepidemiological magnitude of HHV coinfections in HIV-infected key populations are scarce in Tanzania and SSA at large. Generating this information is an essential step towards controlling HIV disease progression and transmission within this group and the general population. Therefore, the present study aimed to determine the seroprevalence of HHV coinfections in HIV-infected key populations in Dar es Salaam, Tanzania.

## 2. Materials and Methods

### 2.1. Study Design

A total of 262 stored serum samples and sociodemographic data (age and sex) from three cross-sectional integrated bio-behavioral surveys (IBBS) conducted in Dar es Salaam, Tanzania, were analyzed [12–14]. The surveys were conducted from November 2017 to January 2018 following the implementation of the national comprehensive HIV intervention package (CHIP) for key populations in 2014. The surveys aimed to investigate HIV prevalence and other sexually transmitted infections (hepatitis B virus and hepatitis C virus infections and syphilis) and associated risk factors among MSM [12], PWID [13], and FSW [14] in the largest metropolitan city of Dar es Salaam.

### 2.2. Study Population

The analyzed samples were a subset of samples obtained from newly diagnosed HIV-infected key populations from the IBBS. The participants whose samples were analyzed included FSW (*n* = 121), MSM (*n* = 97), and PWID (*n* = 44). The present study selected a population of newly diagnosed HIV-infected key populations so as to assess the burden of HHV infections among this population and reduce transmissions across uninfected populations through preventative interventions of sexually transmitted infections in our local settings. In addition, this population is suitable for providing baseline data that will be used in further study that intends to assess the association between CMV and non-AIDS complications. The sociodemographic and behavioral characteristics of the participants have been previously reported [12–14]. The present study obtained ethical approval from the Institutional Review Board (IRB) of the Muhimbili University of Health and Allied Sciences (MUHAS).

### 2.3. Detection of CMV, HSV-1, and HSV-2-Specific IgG and IgM Antibodies

The seroprevalences of HHV (CMV, HSV-1, and HSV-2) were determined by standard methods using commercially available immunoassays. First, we performed a screening test to detect IgG and IgM for CMV, HSV-1, and HSV-2 using an OnSite TORCH Panel Rapid test, CTK Biotech (lot no. P0907N9A00), as per the manufacture's instructions. Thereafter, all samples were confirmed by using the enzyme-linked immunosorbent assay (ELISA). For the detection of CMV, a human anti-CMV IgG/IgM ELISA kit (CMV) (ab108724) (Abcam Inc., United States) was used, and HSV-1 and HSV-2, human anti-HSV 1 and HSV-2 IgG/IgM ELISA kits (ab108737) (Abcam Inc., United States) were used. The ELISA tests were performed as per the manufacturer's instructions and as reported elsewhere [15]. The ELISA microtiter plate reader was used to read the absorbance at 450 nm.

### 2.4. Statistical Analysis

A descriptive analysis was performed where continuous variables were summarized using the median with interquartile range (IQR) and proportions for categorical variables. The association between seroprevalence of HHV coinfections across the key population groups was analyzed by using Fisher's exact test, and a comparison of medians was made by using the Kruskal–Wallis test. All data were analyzed by using GraphPad Prism version 6.07. The statistical significance level was set at *p* < 0.05.

## 3. Results

### 3.1. Study Participants' Characteristics

We analyzed 262 newly diagnosed HIV-infected key populations who were on antiretroviral treatment naïve with the median age of 32 years' interquartile range (IQR) (25–40). We observed a significant difference of median age (*p* < 0.0001) among the key populations; whereby, FSW, MSM, and PWID had the median age of 35 years, IQR (29–40), 25 years, IQR (22–33), and 38 years, IQR (33–43), respectively (data not shown). In addition, gender distribution among PWID was 77.3% (34/44) males and 22.7% (10/44) females. The participants' characteristics are summarized in Table 1.

### 3.2. Seroprevalence of HHV Infections in HIV-Infected Key Populations

**Figure 1:**
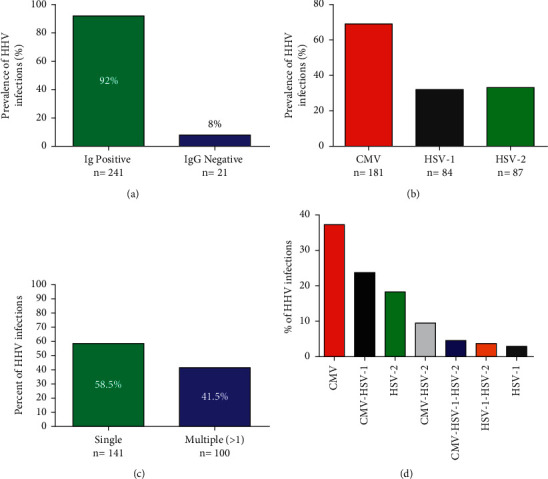
Seroprevalence of HHV coinfections among HIV-infected key population. (a) The overall seroprevalence of human herpesvirus (CMV, HSV-1, and HSV-2) infections (*n* = 262). (b) The overall prevalence of each of the HHV infections. (c) The proportion of single HHV infection and multiple infections (> more than 1 HHV infection) among key populations tested positive for IgG (CMV, HSV-1, and HSV-2). (d) The distribution (frequency) of HHV single and multiple coinfections in HIV-1-infected key populations.

The overall seroprevalence of HHV infections was 92% (95% CI: 87.7–95.3%) (Figure 1(a)) as detected by the IgG/IgM ELISA test. However, we did not detect markers for acute infection (HHV IgM) in any of the tested samples. The seroprevalence of the infections in the descending order of magnitude was CMV 69.1% (181/262), HSV-2 33.2% (87/262), and HSV-1 32.1% (84/262) (Figure 1(b)). Most of the samples (58.5%) tested positive for a single infection, i.e., IgG of either CMV, HSV-1, or HSV-2 (Figure 1(c)). Stratification to determine the contribution of multiple infections (infection with more than one virus) revealed that double infection was present in 36.9%; whereby, CMV-HSV-1 was 23.7% (95% CI: 18.3–29.1%), CMV-HSV-2 was 9.5% (95% CI: 5.8–13.5%), and HSV-1-HSV-2 was 3.7% (95% CI: 1.3–6.1%). Only 4.6% (95% CI: 1.9–7.3%) of the samples were IgG positive for all three infections (CMV, HSV-1, and HSV-2) (Figure 1(d)).

### 3.3. Distribution of HHV Infections among the HIV-Infected Key Populations

**Figure 2:**
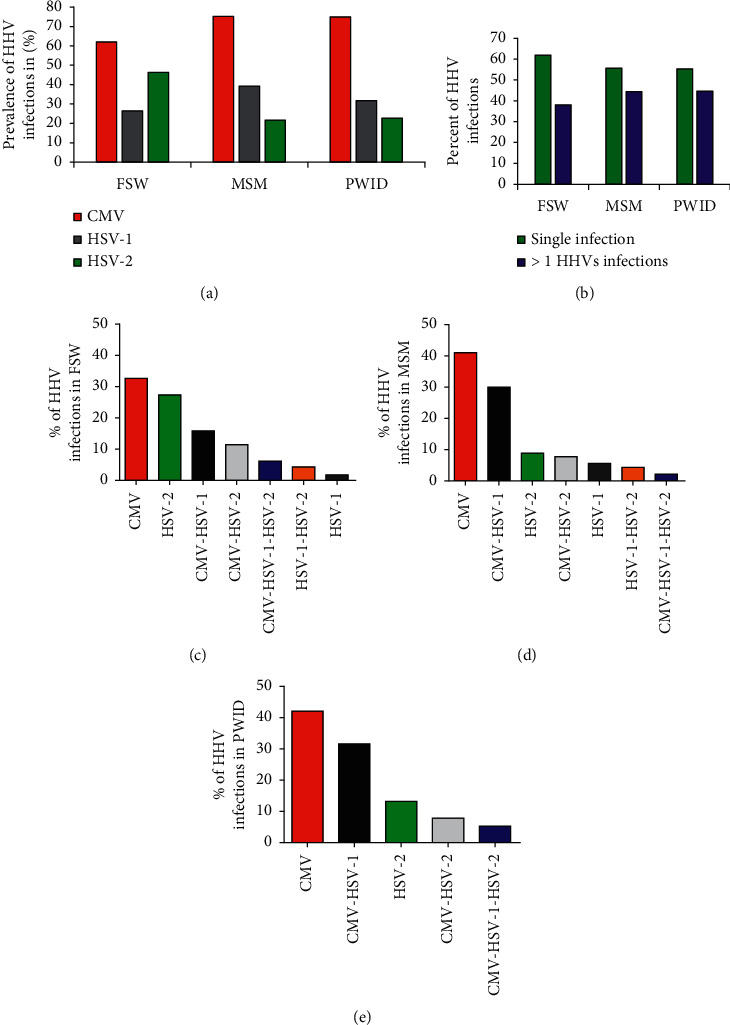
Distribution of HHV coinfections among HIV-infected key population groups. (a) The overall prevalence of HHVs (CMV, HSV-1, and HSV-2) among the key populations FSW (*n* = 121), MSM (*n* = 97), and PWID (*n* = 44). (b) The prevalence of single HHV (either CMV or HSV-1 or HSV-2) infection and multiple infections (> more than 1 HHVs coinfections) across each key population who tested positive for IgG (*n* = 241). Figures indicate the distribution of single and multiple HHV infections among (c) FSW, (d) MSM, and (e) PWID HIV-infected key populations who tested positive for IgG (*n* = 241).

A higher seroprevalence of HSV-2 infection was found among FSW (46.3%) (*p*=0.0001) (Fisher's exact test) compared to PWID (21.6%) and MSM (22.7%). However, there was no statistically significant difference in seroprevalence of CMV infection across the three population groups: 62% (75/121), 75.3% (73/97), and 75% (33/44) among FSW, MSM, and PWID, respectively (*p* > 0.05). HSV-1 seroprevalence was also comparable across key population groups, i.e., 26.4%, 39.2%, and 31.8% for FSW, MSM, and PWID, respectively (*p* > 0.05) (Figure 2(a)).

Single infections were predominant across each key population: FSW (61.9%), MSM (55.6%), and PWID (44.7%) (Figure 2(b)). However, single infections were more observed in the FSW group compared to others (*p*=0.023). CMV was the most predominant single infection in the three key population groups: 32.7% (37/113), 41.1% (37/90), and 42% (16/38) for FSW, MSM, and PWID, respectively (Figures 2(c)–2(d)). Among HHV coinfections, HSV-2 (*p*=0.001) and CMV-HSV-2 (*p*= 0.016) coinfections were common among FSW compared to MSM and PWID subpopulations (data not shown) (Fisher's exact test).

**Figure 3:**
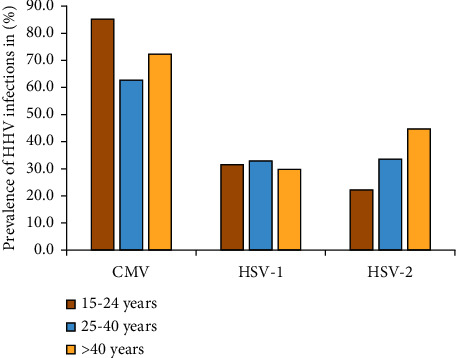
Distribution of HHV coinfections among different age groups in key population groups. The figure depicts the distribution (frequency) of HHVs (CMV, HSV-1, and HSV-2) coinfections among the key populations with reference to age categories.

Analysis of HHV coinfections with HIV according to age groups indicated that HSV-2 prevalence increased with age, whereas we did not find a similar pattern for CMV and HSV-1 (Figure 3). Further stratification revealed that CMV single infection and HSV-1-HSV-2 were significantly more common among adults aged 25–40 (*p*=0.001) and (*p*=0.014), respectively, while CMV-HSV-2 and CMV-HSV-1-HSV-2 infections were significantly more common among those aged >40 years (*p*=0.042) and (*p*=0.006), respectively (data not shown) (Fisher's exact test).

## 4. Discussion

Several studies have provided substantial evidence that HIV coinfection with some HHVs, specifically CMV, HSV-1, and HSV-2, plays a significant role in HIV-1 transmission and acquisition, independently from immunosuppression status and subsequently affect the clinical outcome [16–18]. However, data on the seroprevalence of these conditions are scarce in SSA, including Tanzania. The present study provides important information on the seroepidemiology of HHV coinfections among newly diagnosed HIV-infected key populations living in the largest metropolitan city of Dar es Salaam, Tanzania.

Our study has revealed that CMV, HSV-1, and HSV-2 IgG seroprevalences among HIV-infected key populations (FSW, MSM, and PWID) in Dar es Salaam are significantly high. In addition, the seroprevalences reported herein are comparable to those previously reported in the general population from similar settings [19–22]. The finding suggests that the high endemicity of HHV infection in Tanzania potentially exposes the general and key populations to a similar risk of acquiring these infections. However, we did not detect IgM for any of the three infections consistent with findings from other studies that investigated HHV IgM seroprevalence in HIV-negative population [23]. This is because HHVs infections are often asymptomatic in immunocompetent persons and are not routinely screened in our local settings; hence, this may explain the high detection frequency of IgG in the present cross-sectional study.

Women tend to have an increased risk of acquiring HSV-2 infection than men during penile-vaginal sex [24]. This study found HSV-2 seroprevalence among FSW to be two times higher than that of MSM and PWID. Previous studies conducted in different parts of Tanzania among HIV-negative adolescents and pregnant women have reported HSV-2 seroprevalence to range from 12.5% to 20.5% [23, 25, 26] PWID and MSM reported in this study. Therefore, the observed relatively high seroprevalence among FSW than women from the general population and other key populations suggests increased vulnerability to HSV-2 infection among FSW. Indeed, two different studies conducted in northern Tanzania among high-risk women in the recreation business (bar and hotel workers) found HSV-2 seroprevalence to range from 56.3% to 67% [26, 27], corroborating our estimates of 46.3% in FSW. Therefore, HIV and sexually transmitted infection prevention programs that target FSW should emphasize duo prevention, screening, and treatment of HSV-2 infection. Furthermore, the present study revealed that HHVs infections increased with age, an observation that is consistent with findings from previous studies among key populations in the city and beyond [28–31]. Given the epidemiological synergy between HIV and HSV-2, the high prevalence of HSV-2 underscores the need to scale up preventive efforts to facilitate the 2030 goal of ending the HIV epidemic [2, 32].

The present study found 41.5% of key populations seropositive for two or more HHV coinfections. This relatively high estimate is similar to that observed in the HIV-infected general and key populations in Africa, underscoring the high endemicity of HHV infection in these settings [22, 33]. Further studies are warranted to elucidate the overall impact of HHV coinfection on HIV disease progression, clinical outcome, chronic inflammation, and the emergence of non-AIDS complications among the HIV-infected population in multiple HHV coinfection contexts.

The present study has some limitations: first, the data presented here are from key populations recruited from one region in Tanzania. However, the data are consistent with HHV seroprevalences reported elsewhere. Second, the study design used in this study was a cross-sectional survey, so it was not possible to observe HHV recurrency and the correlation of HHVs infection with HIV disease progression and clinical outcomes. Further studies are necessary to investigate the interaction between HHV and HIV coinfections.

## 5. Conclusion

Our study revealed high IgG seroprevalence of HHV coinfections among key populations. These findings are alarming because they suggest that there may be a risk for recurrence of HHV infections, and also, IgG seropositive individuals may harbor replicating viruses that subsequently may enhance HIV replication and disease progression in this population. Therefore, these findings warrant the need for public health prevention and intervention programs for HHV infection among key populations in our setting. Additional emphasis on controlling HSV-2 infection should be considered in prevention strategies targeting FSW to achieve the 2030 goal of ending the HIV epidemic.

## Figures and Tables

**Table 1:** Summary of HIV-infected key populations characteristics.

Key population group	FSW		MSM		PWID	
Age						
Median (IQ)	35 (29–40)		25 (22–33)		38 (33–43)	
	Number (*n*)	%	Number (*n*)	%	Number (*n*)	%
15–24	13	10.7	40	41.2	1	2.3
25–40	80	66.1	52	53.6	29	65.9
>40	28	23.1	5	5.2	14	31.8
Total	121	100	97	100	44	100

Sex						
Female	121	100			10	22.7
Male			97	100	34	77.3

## Data Availability

The data used to support the findings of this study are available from the corresponding author upon request.
